# Effectiveness of bevacizumab in the treatment of metastatic colorectal cancer: a systematic review and meta-analysis

**DOI:** 10.1186/s12876-024-03134-w

**Published:** 2024-02-01

**Authors:** Yu Song, Qianqian Mao, Manling Zhou, Cheng-Jiang Liu, Li Kong, Ting Hu

**Affiliations:** 1 Department of Intensive Care Unit, the First Affiliated Hospital of Shandong Traditional Medical University, 250000 Jinan, China; 2 Department of Oncology, School of Medicine, Zhongda Hospital, Southeast University, No. 87 Dingjiaqiao Road, 210009 Nanjing, Jiangsu Province China; 3 Department of Oncology, Shenzhen Longhua District Central Hospital, Shenzhen, China; 4 Department of General Medicine, Affiliated Anqing First People’s Hospital of Anhui Medical University, 246000 Anqing, AnHui China; 5 Department of General Practice, Anqing Municipal Hospital, 246000 Anqing, AnHui China

**Keywords:** Bevacizumab, Colorectal cancer, Odds ratio, Combination

## Abstract

**Objective:**

To evaluate the benefit of bevacizumab under the comprehensive treatment strategy and its advantages over other drugs, so as to provide reference for the formulation of clinical plans.

**Methods:**

As of October 1, 2022, the randomized controlled clinical trials of bevacizumab in combination with metastatic colorectal cancer published in PubMed, Cochrane Library and Medline databases were searched. The odds ratio (OR) and its 95% confidence interval (CI) were used to evaluate the short-term disease control effect and long-term survival of the treatment strategy.

**Results:**

21 RCTs (6665 patients; 3356 patients in the experimental group and 3309 patients in the control group; average age, 55–75 years) were treated with bevacizumab as the experimental group for metastatic colorectal cancer. BEV has stronger anti-tumor activity than the single treatment scheme (OR = 1.30, 95% CI: 1.11–1.52). And Benefits of the BEV group were 0.73 (0.55, 0.96), 1.26 (0.71, 2.24), 1.63 (0.92, 2.87) and 0.07 (0.02, 0.25) compared with CET, VAN, CED and PAN respectively. The disease control of BEV combined therapy was better (OR = 1.36, 95% CI: 1.04–1.78). The same as compared with cediranib (OR = 1.94, 95% CI: 1.06–3.55). However, the long-term prognosis of BEV, including the overall survival (HRs = 0.98, 95% CI: 0.84–1.15) and progression-free survival (HRs = 1.05,95% CI: 0.97–1.13) were not prolonged. The survival benefits of cetuximab and panitumumab were not reflected.

**Conclusion:**

The addition of BEV can enhance the anti-tumor ability and disease control, while cetuximab and panitumumab may have stronger ability. However, it did not effectively improve the survival of patients. A more reasonable and effective treatment plan needs more clinical experimental support.

**Supplementary Information:**

The online version contains supplementary material available at 10.1186/s12876-024-03134-w.

## Introduction

Colorectal cancer is one of the most common malignant tumors of digestive tract in the world [1]. More than 1.8 million cases of colorectal cancer were diagnosed in 2018, making it the third most common cancer in the world, accounting for 10% of all cancer diagnoses [2]. Nearly 25% of colon cancer is diagnosed as stage II in western countries [3]. Over the past 20 years, great progress has been made in the treatment of colorectal cancer. The median survival of patients with metastatic colorectal cancer (mCRC) receiving multimodal therapy can reach 30 or more months, but the prognosis of patients with metastatic colorectal cancer still need to be improved [4, 5].

For a long time, the choice of first-line treatment plan was a key step in the treatment route of every patient with mCRC. In the past few years, the combination of dual chemotherapy drugs (fluoropyrimidine plus irinotecan or oxaliplatin) and targeted drugs were the first choice for most patients [6]. In a multi-center international study, it was evaluated that the 5-year and 10-year overall survival rates (OSR) of patients with high-risk phase II CC were 88% and 75%, respectively, when oxaliplatin was added on the basis of fluorouracil and under the adjuvant folic acid, fluorouracil and oxaliplatin (FOLFOX) chemotherapy [7, 8]. Several promising therapeutic methods including chemotherapy and molecular agents targeting epidermal growth factor receptor (EGFR) and vascular endothelial growth factor (VEGF) have been reported [9–12]. These reports suggested that targeted drug combination chemotherapy can improve the rate and efficiency of hepatectomy, thereby improving the progression-free survival (PFS) and overall survival (OS) of patients.

Bevacizumab, an anti human vascular endothelial growth factor, was subsequently approved to be combined with fluorouracil in the treatment of patients with metastatic colorectal cancer [13, 14]. Bevacizumab could increase the activity of multi-drug therapy and fluoropyrimidine monotherapy in the case of metastasis. Advantage of bevacizumab combined with chemotherapy in metastatic colorectal cancer patients may be due to the increased sensitivity of tumor cells to chemotherapy, or the better distribution of chemotherapy drugs in tumors [14–16]. But its front-line cooperation with FOLFOX and other drugs has been questioned [17]. The comparison between BEV to placebo and BEV to CET and PAN may indeed confused. Therefore, we aim to conduct a meta-analysis of randomized controlled trials (RCTs) of bevacizumab combined with multiple protocols for the treatment of metastatic colorectal cancer, and compare the efficacy of bevacizumab compared with placebo or other drugs for the treatment of metastatic colorectal cancer, so as to provide more optional basis for the clinical treatment of metastatic colorectal cancer.

## Materials and methods

### Search Strategy

We follow the Preferred Report Item for Systematic Reviews and Meta-analyses statement to perform the meta-analysis. As of October 1, 2022, we have conducted a systematic search on medical databases (PubMed, Medline and Cochrane Library). Language was restricted English only. The following search keywords were used: “Colorectal Neoplasm”, “Colorectal Tumors”, “Colorectal Cancers”, “Rectal Neoplasms” and “Bevacizumab”, “Antineoplastic Chemotherapy Protocol”, “Antineoplastic Drug Combinations”, “Combined Antineoplastic Agent”. We also searched the bibliography of confirmed reports for more references. Two researchers jointly completed this search process.

### Inclusion and exclusion criteria

The inclusion criteria were as follows:

- Randomized controlled trials;
- Adult patients with mCRC confirmed histologically or cytologically (age > = 18 years);
- The experimental group was assigned a combination of bevacizumab therapy, control group was allocated placebo or other;
- Basic characteristics for patients were described, and the primary outcome were ORR, CRR, DCR, OS, PFS, etc.

The exclusion criteria were as follows:

- Patients who had received adjuvant therapy in the first month of grouping were excluded;
- History of stroke, transient ischemic attacks, myocardial infarction/unstable angina, significant peripheral vascular disease, bleeding diathesis, uncontrolled hypertension, grade > 1 neuropathy, and allergy to platinum compounds;

(c) Animal experiments, reviews, abstracts, reviews, reports;

(d) Specific patient population (elderly or patients with liver metastasis only) or treatment methods (induction therapy with anti EGFR (epidermal growth factor receptor) antibody).

### Data extraction and Quality Assessment

Screening and data extraction were conducted in duplicate by two investigators (Hu and Liu) independently. Any differences were resolved through discussion or consultation with other researchers. The data was wxtracted including name of the first author, year of publication, sample size, average age of patients, disease stage, intervention and control strategies, follow-up and main results such as objective response rate (ORR), disease control rate (DCR), overall survival (OS) and median progression-free survival (PFS). For those studies that did not give a specific effect value, it was calculated based on the effect and number of people given the treatment. In order to get enough information, we downloaded the full text. If in doubt, please ask the original author for help. Jadad scale, which assessing data related to randomization, blinding, and study withdrawal, was used to evaluate the methodological quality of selected randomized clinical trials [18]. The evaluation content of the scale are generation of random sequence, randomized hiding, blind methods and withdrawal and loss of interview. Full score of a randomized controlled trial was 7. Randomized controlled trials with scores > = 4 were considered to be of good quality and can be included in the meta-analysis.

### Statistical analysis

Based on the recommendations of the Cochrane collaboration, quantitative synthesis of the indicators were included in the study. The data was pooled by conducting meta-analysis. If data were allowed, Stata 16.0 software(Stata Corporation, College Station, TX) would be used. We summarized the main results of the experimental group and the control group to obtain the efficacy of bevacizumab in multiple comprehensive therapies. Random-effects model was used for meta-analysis considering potential sources of clinical heterogeneity. When I^2^ ˃ 50%, subgroup analysis based on baseline, intervention and/or sensitivity analysis eliminate studies one by one would be conducted to explore the source of heterogeneity [19, 20]. Small sample effect and publication bias were detected by funnel plots and statistical tests, respectively.

## Results

### Search results

**Fig. 1:**
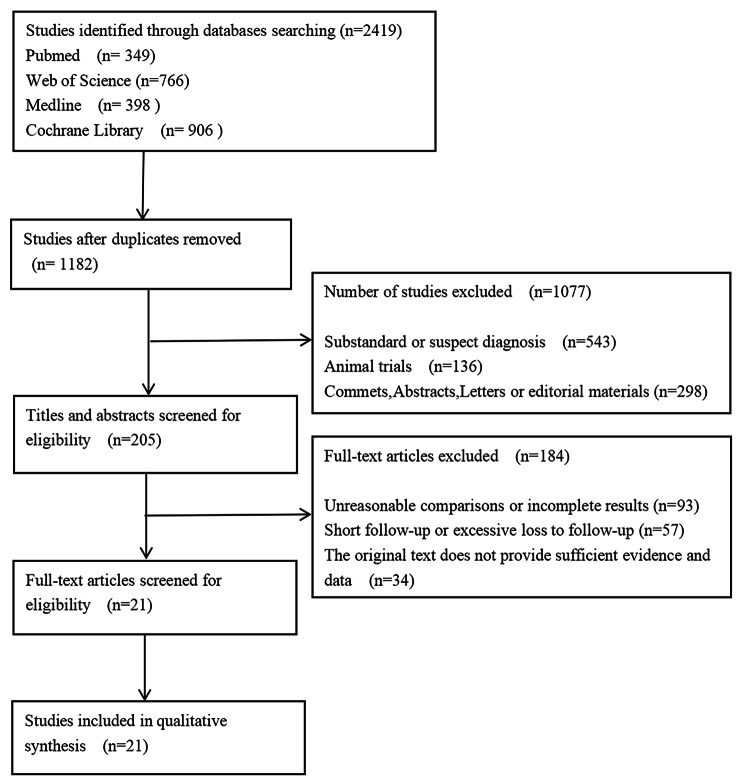
Screening flow chart of included studies

According to the pre-screening strategy, 21 randomized clinical trial were included in this study by two researchers [14, 21–40]. Figure 1 showed the whole process of selecting documents. The main characteristics of each study were summarized in Table 1. There were 6665 participants, including 3356 in the experimental group and 3309 in the control group. Their overall average age was between 55 and 75 years. We also checked the gender ratio, Tumor site (colon/rectum), BRAS mutation status (mutant/wild type) and ECOG (Eastern Cooperative Oncology Group) status (An important index for evaluating the general health status of tumor patients) of the selected personnel, and identified the comparability. FOLFIRI scheme and FOLFOX scheme were the most commonly used schemes, in addition to CAP and CT therapy. The experimental group only added appropriate dose of bevacizumab on the basis of the control group. In addition, cetuximab (CET), vanucizumab (VAN), cediranib (CED), panitumumab (PAN) were also used as the control group for comparison with bevacizumab (Table 2). Supplement Table 1 showed all results of our evaluation on the methodological quality of the randomized clinical trials. Randomization concealment, loss of interview and withdrawal were their main defects. However, Jadad rated them all at or above 4. Therefore, they entered the meta comprehensive analysis.

**Table 1:** The characteristic of included studies

Study ID	Group	Age(years)	Sex (male/female)	ECOG status(0/≥1)	Regimen/Dose	Sample	Tumor site (colon/rectum)	BRAS mutation status (mutant/wild type)	Main outcomes/endpoint
Salazar 2015^[21]^	Experience	64(37–77)	25/19	22/22	capecitabine + BEV(5 mg/kg)	44	NA	NA	pCR,safety
	Control	60(42–78)	30/16	30/16	capecitabine	46	NA	NA	
Salazar 2020^[22]^	Experience	64(37–77)	25/19	22/22	capecitabine + BEV(5 mg/kg)	44	NA	NA	YpCR,DFS,DRFS,OS
	Control	60(42–78)	30/16	30/16	capecitabine	46	NA	NA	
Qin 2021^[23]^	Experience	56.7 ± 11.6	214/123	116/221	BEV	337	254/83	152/160	PFS,ORR
	Control	57.4 ± 11.2	190/148	110/228	HLX04	338	251/87	141/171	
Oki 2019^[24]^	Experience	64(32–80)	34/23	51/6	mFOLFOX6 + BEV(5 mg/kg)	57	48/9	NA	PFS,RR
	Control	65(42–79)	34/25	51/8	mFOLFOX6 + CET	59	45/14	NA	
Heinemann 2020^[25]^	Experience	64(31–76)	121/62	98/85	FOLFIRI + BEV(5 mg/kg)	183	NA	NA	ORR,PFS,OS
	Control	65(41–76)	128/41	94/75	FOLFIRI + CET	169	NA	NA	
Fischer 2022^[26]^	Experience	18–75	133/68	109/92	FOLFIRI + BEV	201	NA	177/24	ORR,PFS,OS
	Control	18–75	146/53	106/93	FOLFIRI + CET	199	NA	175/22	
Stathopoulos 2010^[27]^	Experience	67(45–82)	73/41	85/29	FOLFIRI + BEV(7.5 mg/kg)	114	NA	NA	RR,OS,toxicity
	Control	62(30–87)	68/40	78/30	FOLFIRI	108	NA	NA	
Chibaudel 2020^[28]^	Experience	57.1(46.0-66.2)	99/95	160/33	FOLFOX4 + BEV (5 mg/kg) for 24 weeks	194	NA	NA	DFS,OS
	Control	57.2(48.2–66.9)	124/68	161/29	FOLFOX for 24 weeks	192	NA	NA	
Bendell 2019^[29]^	Experience	63.0(29–81)	38/47	47/48	BEV combined with mFOLFOX6 for 8cycles	95	77/18	45/36	PFS,ORR,OS
	Control	64.0(27–82)	56/38	60/34	VAN 2000 mg(q2w) combined with mFOLFOX6 for 8cycles	94	73/21	37/43	
Chakravarthy 2020^[30]^	Experience	53.9 ± 9.9	112/67	109/70	mFOLFOX6 + BEV(5 mg/kg) for 9 cycles	179	NA	NA	OS,DFS,AEs,HRQoL
	Control	54.3 ± 11.7	114/62	106/70	mFOLFOX6 for 9 cycles	176	NA	NA	
Cunningham 2013^[31]^	Experience	NA	39/27	48/18	mFOLFOX6 + BEV(10 mg/kg)	66	38/28	NA	PFS,OS,ORR
	Control	NA	47/26	44/29	mFOLFOX6 + CED 20 mg	71	50/21	NA	
	Control	NA	49/22	42/29	mFOLFOX6 + CED 30 mg	73	51/22	NA	
Shitara 2016^[32]^	Experience	64(26–78)	39/19	43/15	FOLFIRI + BEV(5 mg/kg)	58	NA	NA	OS,PFS,ORR,safety
	Control	62(31–82)	34/25	47/12	FOLFIRI + PAN	59	NA	NA	
Snoeren 2017^[33]^	Experience	62(57–70)	NA	17/6	capecitabine + BEV	39	13/14	NA	DFS,OS,toxicity
	Control	61(53–63)	NA	13/11	capecitabine	38	17/13	NA	
Aparicio 2018^[34]^	Experience	80.9(75.2–88.3)	26/25	NA	BEV combined with CT, at least 6 months	51	37/14	NA	ORR,PFS,OS,
	Control	80.1(75.0-90.6)	30/21	NA	Individual CT, at least 6 months	51	37/14	NA	
Hurwitz 2004^[35]^	Experience	59.5	237/165	233/165	Irinotecan + Fluorouracil + Leucovorin + BEV(5 mg/kg)	402	310/92	NA	OS,PFS,ORR
	Control	59.2	247/164	226/185	Irinotecan + Fluorouracil + Leucovorin	411	333/78	NA	
Sharf 2022^[36]^	Experience	58	10/9	14/5	CI + BEV	19	13/3	NA	PFS,OS,ORR,
	Control	54	10/7	12/5	CI	17	10/5	NA	
Venook 2017^[37]^	Experience	59.0(21.8–85.0)	348/211	324/235	CT + BEV(5 mg/kg)	559	334/142	NA	OS,PFS,RR
	Control	59.2(20.8–89.5)	349/229	333/245	CT + CET	578	355/138	NA	
Passardi 2015^[38]^	Experience	66(34–83)	108/68	144/32	CT + BEV	176	135/41	64/91	OS,PFS,ORR
	Control	66 (33–82)	115/79	154/40	CT	194	143/51	98/71	
Dotan 2012^[39]^	Experience	59(45–78)	8/4	3/9	capecitabine + oxaliplatin + cetuximab + BEV(7.5 mg/kg)	12	NA	3/7	RR,TTP,OS
	Control	58(42–74)	10/1	10/1	capecitabine + oxaliplatin + cetuximab	11	NA	2/8	
Moehler 2009^[40]^	Experience	60(37–80)	22/7	25/4	CAPIRI + BEV	29	14/15	NA	RR,toxicity,PFS,OS
	Control	66(55–81)	9/8	12/5	CAPIRI	17	10/7	NA	
Cremolini 2016^[41]^	Experience	29–75	150/102	227/25	FOLFIRI + BEV(5 mg/kg)	252	189/63	NA	PFS,OS,RR
	Control	27–75	75/47	85/37	FOLFIRI	122	81/41	NA	

Note: NA means not available

**Table 2:** Effect and prognosis of colorectal cancer patients compared to BEV addition

Outcomes	Control	Number of study	Effect and 95%CI	I^2^	P
ORR	Placebo	4	0.73(0.55,0.96)	42.7%	0.155
	VAN	1	1.26(0.71,2.24)	-	-
	CED	2	1.63(0.92,2.87)	0%	0.922
	PAN	1	0.07(0.02,0.25)	-	-
DCR	Placebo	4	1.13(0.77,1.66)	0%	0.941
	CET	2	1.71(0.97,3.00)	0%	0.320
	CED	2	1.94(1.06,3.55)	0%	0.374
	PAN	1	0.92(0.36,2.31)	-	-
OS	Placebo	6	1.13(0.91,1.42)	56.2%	0.044
	CET	3	0.84(0.75,0.94)	0%	0.511
	PAN	1	0.86(0.56,1.32)	-	-
PFS	Placebo	5	1.20(1.06,1.37)	0%	0.690
	CET	3	0.93(0.83,1.04)	0%	0.752
	CED	2	1.22(0.91,1.64)	0%	0.764
	PAN	1	0.88(0.60,1.29)	-	-

### Objective remission rate (ORR)

**Fig. 2:**
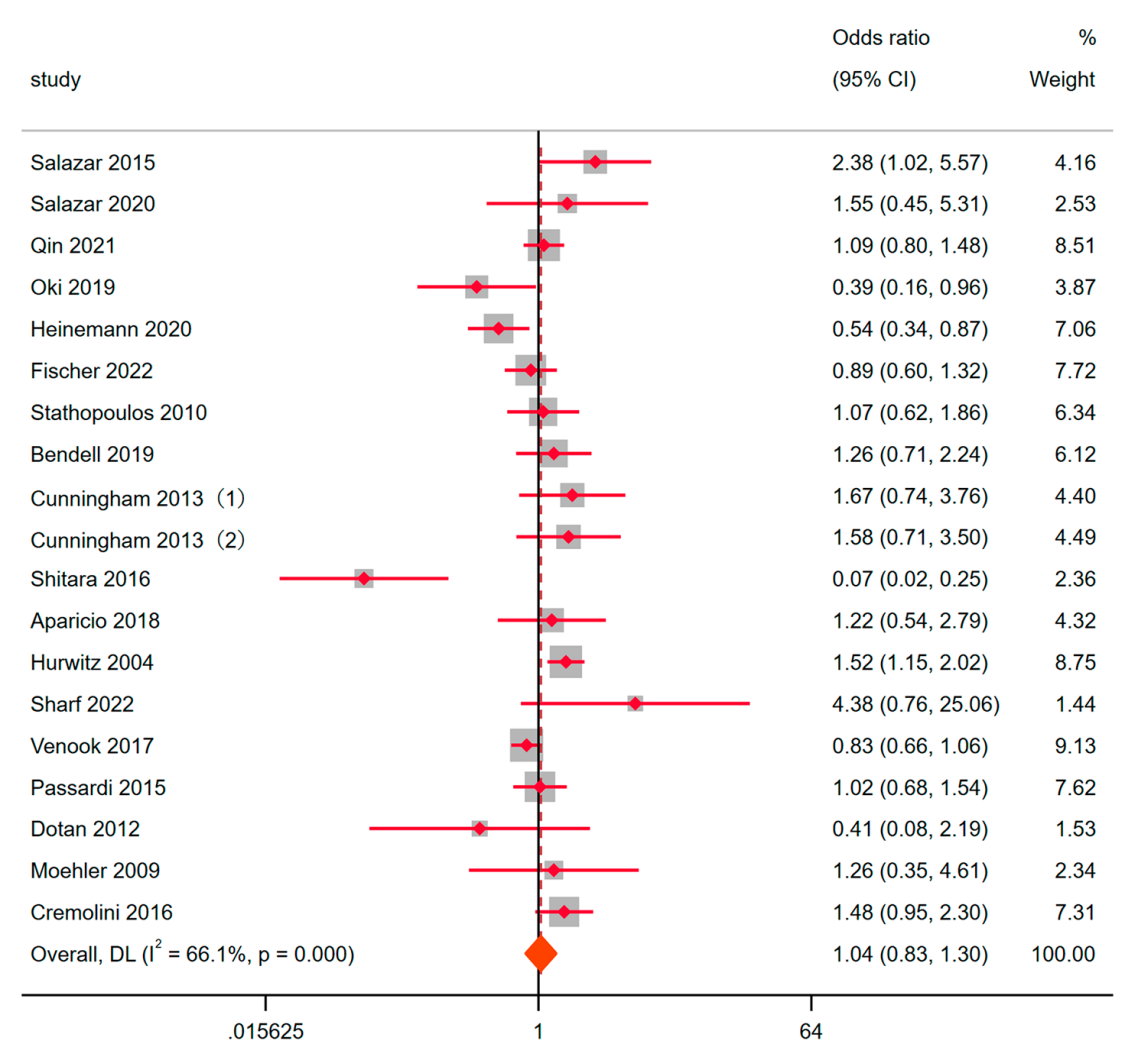
ORR to the combination of bevacizumab for Metastatic colorectal cancer

**Fig. 3:**
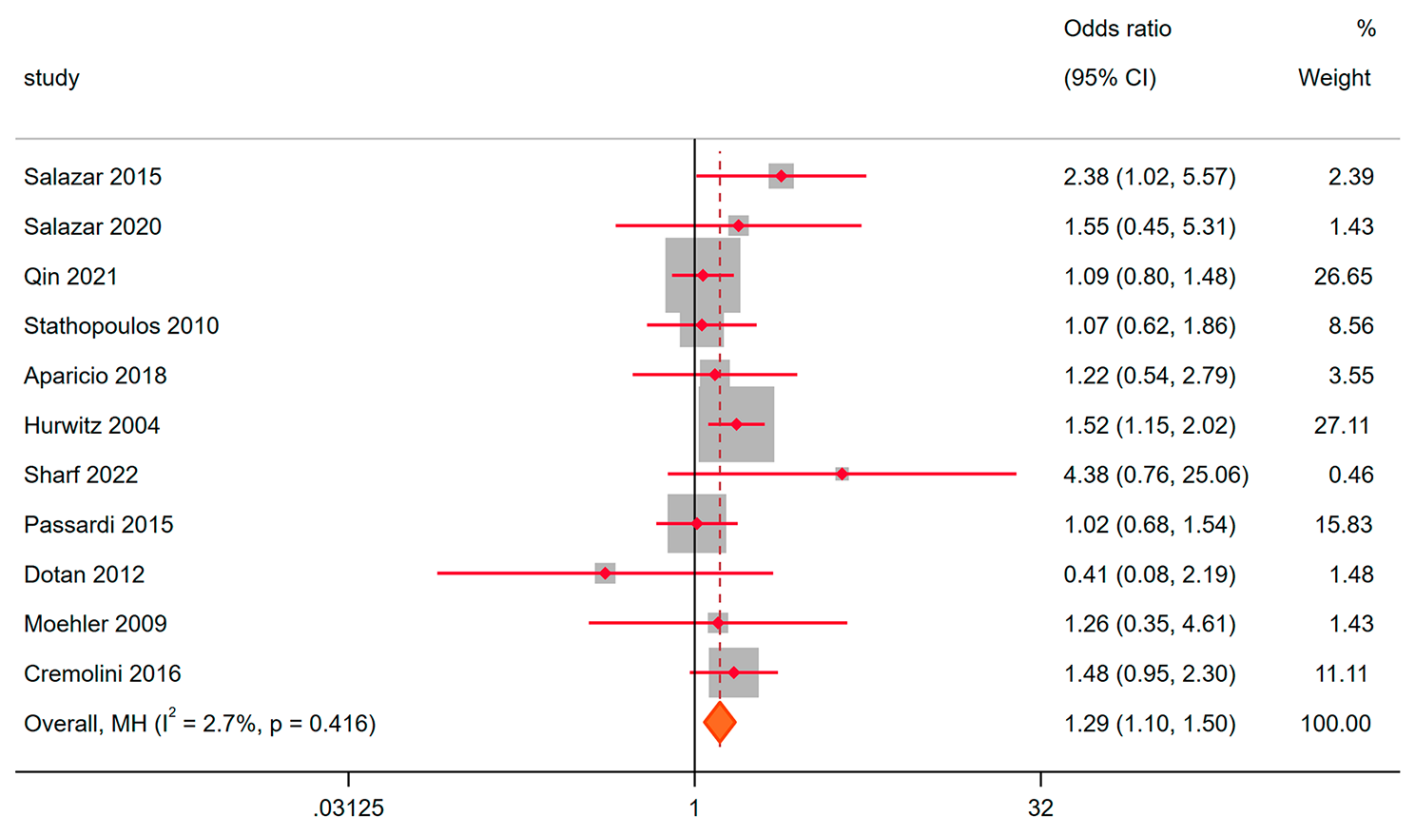
ORR to the bevacizumab combination compared with placebo for Metastatic colorectal cancer

18 randomized clinical trials described objective remission rates for metastatic colorectal cancer in the experimental and control groups. Heterogeneity analysis showed that I^2^ = 66.1%, *P* < 0.05. Random effect model was used for meta-analysis. OR = 1.04, 95% CI: 0.83–1.30, *P* = 0.747 (Fig. 2). This showed that the experimental group only adding bevacizumab did not have complete advantages, which enhanced the anti-tumor activity of the comprehensive treatment scheme. Subsequently, we used a fixed-effect model to pool estimates of those placebo-controlled studies except when significant heterogeneity was found according to a random-effects model. The results of fixed effects showed that OR = 1.29, 95% CI: 1.10–1.50, *P* = 0.001 (Fig. 3). Advantage was found in the experimental group. It was worth noting that the study of Dotan was confirmed that the experimental group was a double antibody group (BEV + CET) with poor therapeutic effect. After removing this study, OR = 1.30, 95% CI: 1.11–1.52, *P* = 0.001. This showed that bevacizumab can indeed enhance the anti-tumor activity of drug therapy.

**Fig. 4:**
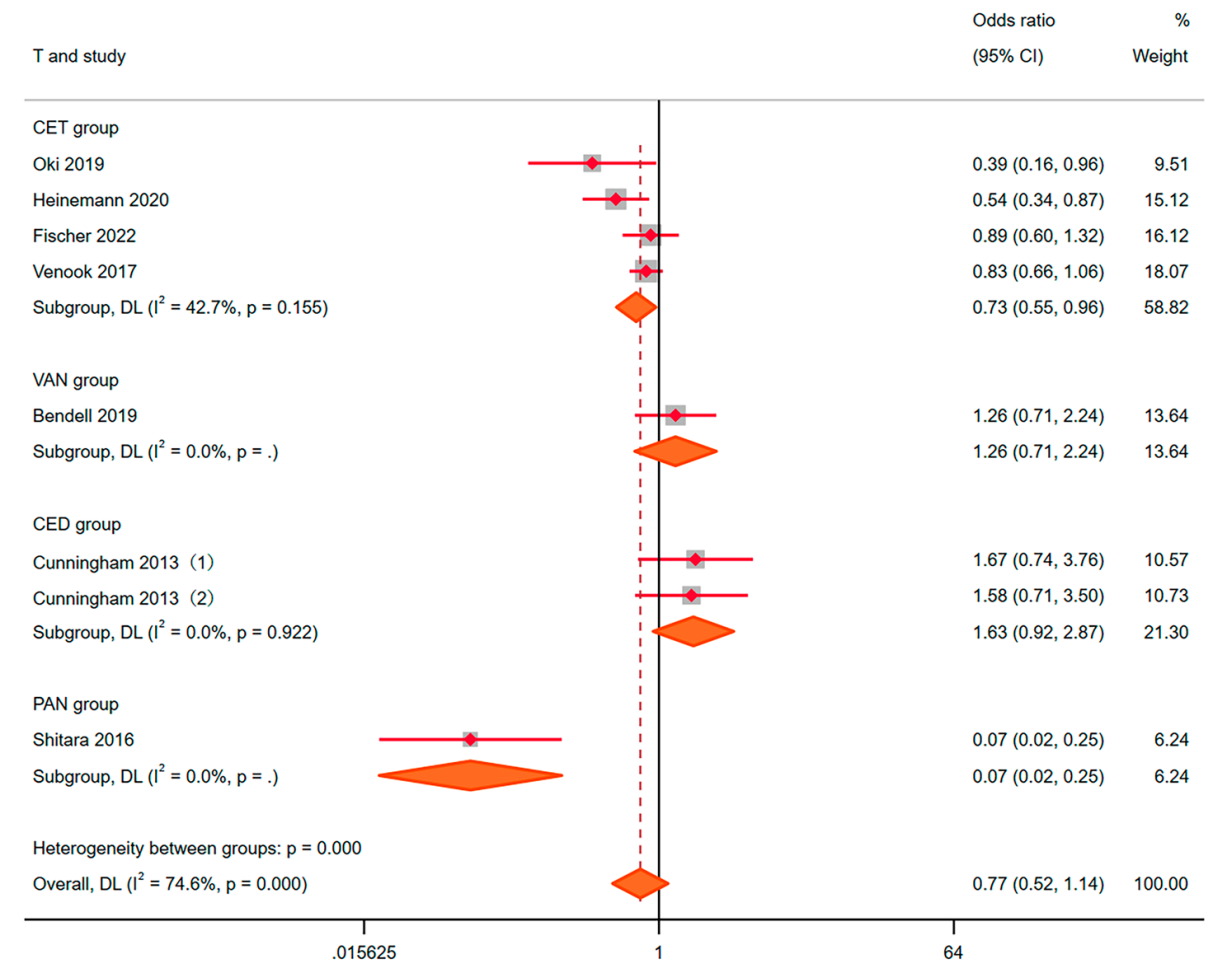
ORR to the bevacizumab combination compared with other drugs for Metastatic colorectal cancer

We also conducted a meta-analysis of studies that were controlled by other drugs. The results of random effects showed that OR = 0.77, 95% CI: 0.52–1.14, *P* = 0.186 (Fig. 4). No advantage of the experimental group was found. Benefits of the BEV group were 0.73 (0.55, 0.96), 1.26 (0.71, 2.24), 1.63 (0.92, 2.87) and 0.07 (0.02, 0.25) compared with CET, VAN, CED and PAN respectively. It meant that the addition of CET and PAN is more effective than that of BEV alone. No advantages were found in VAN and CED compared to BEV. We also used fixed effect model and random effect model for mutual verification to ensure the stability and accuracy of the above analysis results. Egger’s test showed that there was no publication bias (*P* > 0.05).

### Disease control rate (DCR)

**Fig. 5:**
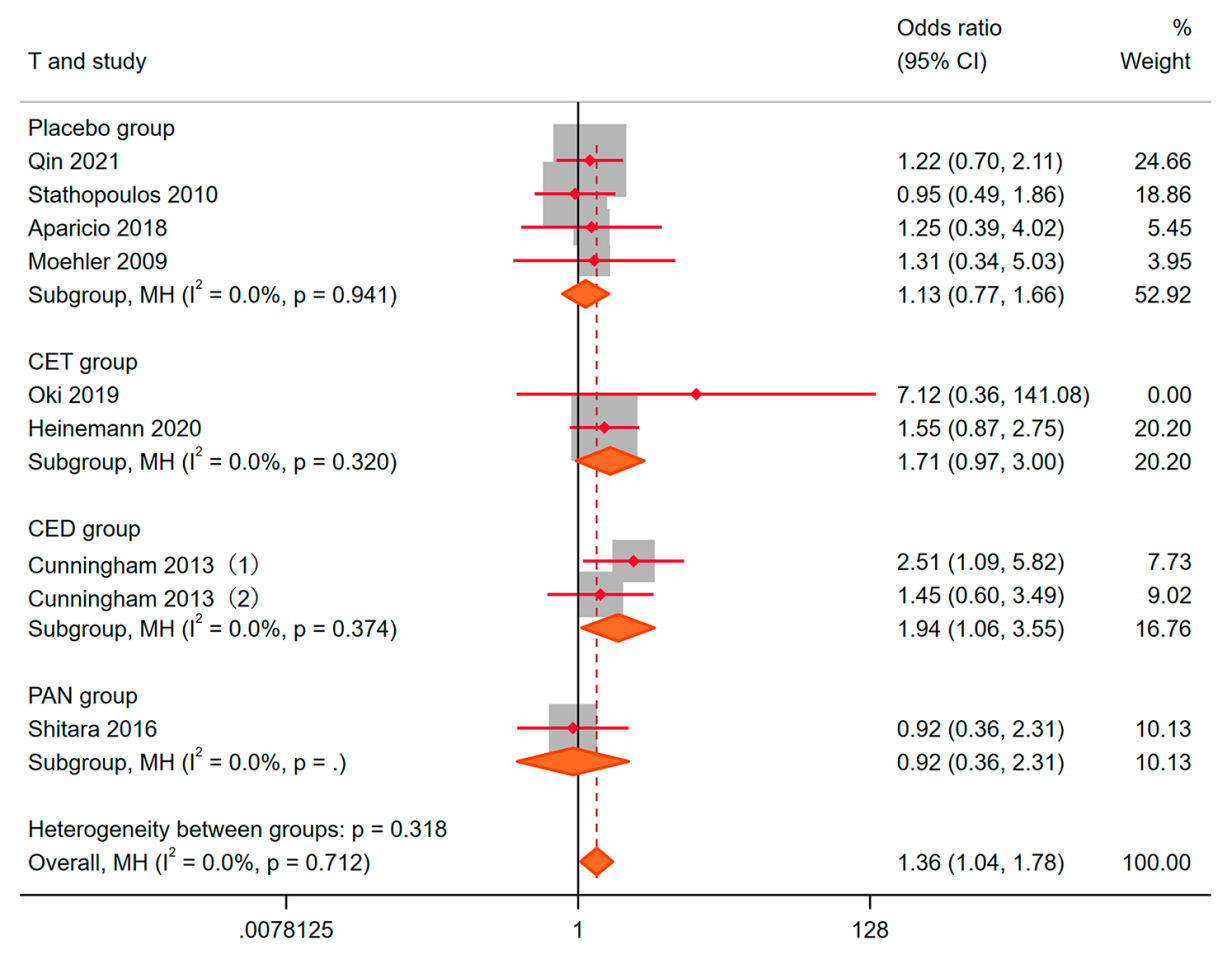
DCR to the bevacizumab combination compared with other drugs for Metastatic colorectal cancer

Data on disease control rates from eight studies were extracted for meta-analysis. The fixed effect model was used for fitting (I^2^ = 0%, *p* = 0.712). OR = 1.36, 95% CI: 1.04–1.78, *P* = 0.024 (Fig. 5). The experimental group added bevacizumab performed better than the control group in disease control. Benefits of BEV were 1.13 (0.77, 1.66), 1.71 (0.97, 3.00), 1.94 (1.06, 3.55) and 0.92 (0.36, 2.31) compared with Placebo, CET, CED and PAN. This meant that The addition of BEV is better than the addition of CED. However, the impact of PAN, CET and BEV on the anti-tumor ability was not different compared to the addition of BEV. The use of random effects models mutually confirms the above conclusions. No publication bias and small sample bias were found through statistical tests.

### Overall survival(OS)

**Fig. 6:**
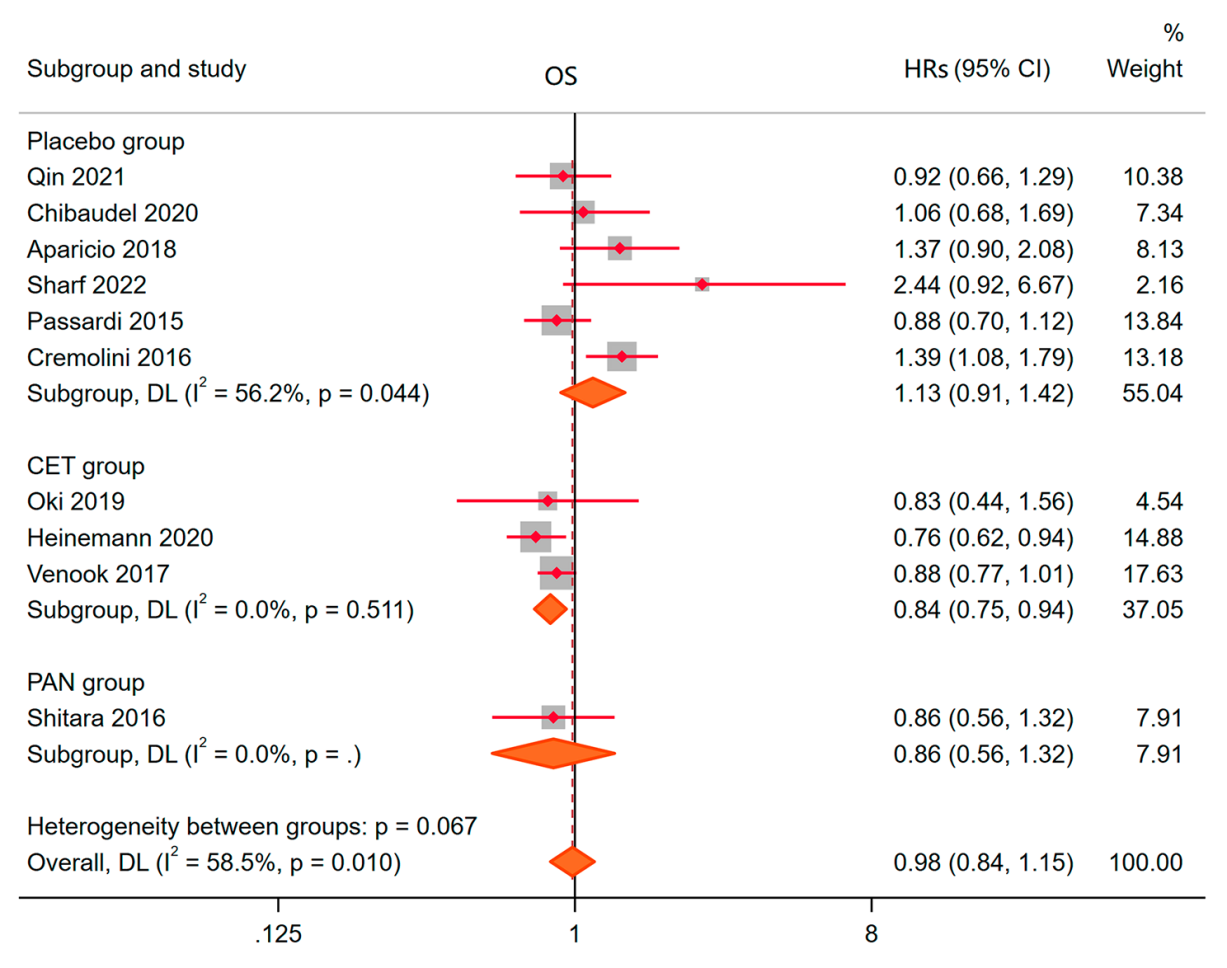
OSR to the bevacizumab combination compared with other drugs for Metastatic colorectal cancer

11 studies described differences in overall survival between experimental and control groups. Heterogeneity analysis showed that I^2^ = 58.5%, *P* = 0.010. Therefore, the random effect model was used to fit the final results. HRs = 0.98, 95% CI: 0.84–1.15, *P* = 0.822, Fig. 6. This indicated that the total survival period has not been prolonged by the addition of BEV. HRs of placebo, CET and PAN as controls were 1.13 (0.91, 1.42), 0.84 (0.75, 0.94) and 0.86 (0.56, 1.32), respectively. This meant that CET had a longer overall survival than BEV alone, and BEV was not better than placebo and PAN. No publication bias and small sample bias were found through statistical tests.

### Progression-free survival (PFS)

**Fig. 7:**
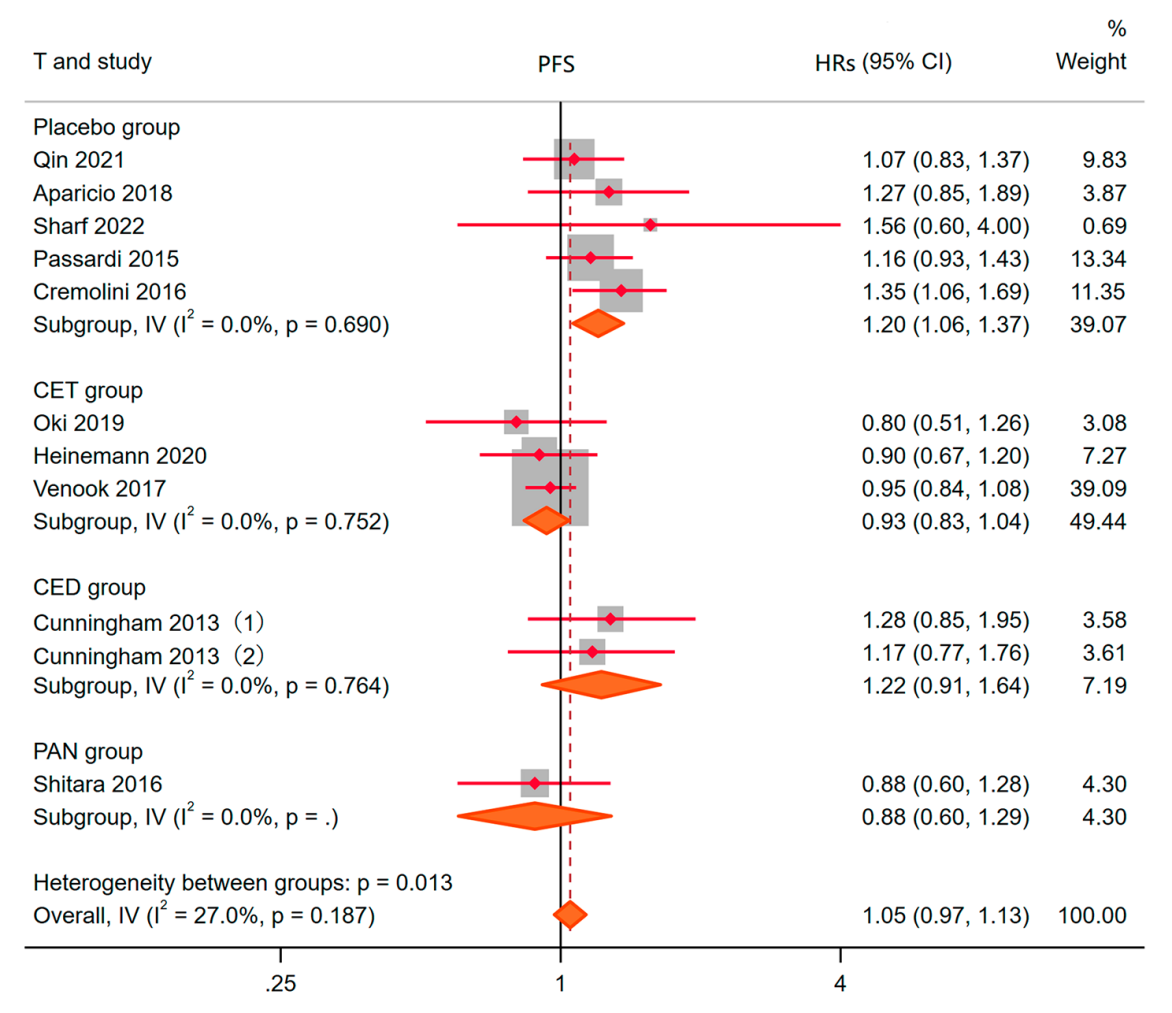
PFSR to the bevacizumab combination compared with other drugs for Metastatic colorectal cancer

10 studies described differences in progression-free survival between experimental and control groups. Heterogeneity analysis showed that I^2^ = 27.0%, *P* = 0.187. Therefore, the fixed effect model was used to fit the final results. HRs = 1.05, 95% CI: 0.97–1.13, *P* = 0.238, Fig. 7. This indicated that the addition of BEV did not prolong the progression-free survival. HRs of placebo, CET, CED and PAN as controls were 1.20 (1.06, 1.37), 0.93 (0.83, 1.04), 1.22 (0.91, 1.64) and 0.88 (0.60, 1.29), respectively. This meant that the addition of BEV will prolong the progression-free survival. There was no difference between CED and PAN compared with CET.

### Sensitivity analysis

Some of the included research methodologies had low quality evaluation. It was restricted that studies with a study quality ≥ 5 can be included in the meta-analysis as sensitivity analysis. Hazard ratio for objective remission rate and disease control rate were OR = 1.00 (95% CI: 0.80–1.24) and OR = 1.55 (95% CI: 1.13–2.12) respectively. Which was consistent with the initial research results. The addition of BEV made disease control profitable. We also analyzed the hazard ratio for overall survival and progression-free survival, OR = 0.89 (95% CI: 0.80–0.99) and OR = 1.02 (95% CI: 0.94–1.11) respectively. This even meant that the addition of BEV cannot improve the overall survival period. There was no difference between fixed effect and random effect models. These results are recorded in the supplementary materials.

## Discussion

Chemotherapy has become the first choice of treatment when colorectal cancer can not be eradicated or distant metastasis occurs [41]. However, it is said that only 30% of patients receiving chemotherapy can achieve the desired effect, and most patients often have poor prognosis [42]. Therefore, it is inevitable for clinicians to choose more beneficial treatment strategies. BEV is the first monoclonal antibody used to treat metastatic colorectal cancer, which can specifically bind VEGF to inhibit the production of vascular endothelial growth [43, 44]. Previous studies have shown that FOLFOX + BEV treatment strategy is superior to a single FOLFOX strategy [45]. However, the benefit of bevacizumab in a broader comprehensive treatment scheme was not clear.

In this study, we found that the anti-tumor activity of BEV added to the comprehensive treatment strategy does not always occupy an absolute advantage (OR = 1.04, 95% CI: 0.83–1.30). As the first certified monoclonal antibody, bevacizumab has certain advantages. Bevacumab maintained its benefits under any treatment regimen (OR = 1.29, 95% CI: 1.10–1.50). It should be noted that the treatment strategy of double antibody is not recommended (OR = 0.41, 95% CI: 0.08–2.19).Trial was forced to stop due to the continuous progress of tumor. In our study, we found that compared with bevacizumab, cetuximab (OR = 0.73, 95% CI: 0.55–0.96) and panitumumab (OR = 0.07, 0.02–0.25) were more effective in anti-tumor treatment. In particular, cetuximab can enhance the efficacy of irinotecan and radiotherapy in experimental systems [46]. On the basis of this, paniximab has potential therapeutic value [47–49]. However, the dosage of bevacizumab is different even under different treatment strategies. This makes the use and benefit of antibody controversial. The same is true for the ability to control disease progression, and bevacizumab still has great benefits (OR = 1.36, 1.04–1.78). Even in the face of cetuximab and paniximab, it is not inferior.

The marginal benefit of combination with oxaliplatin, which has no effect on PFS but no effect on OS, seems to be applicable to other trials involving VEGF inhibitors. Our research evidence may reinforce the impression that oxaliplatin may not be an ideal partner for such target inhibitors, which is similar to the results of the recent two studies [50, 51]. Therefore, the available data are insufficient to draw a conclusion on whether the addition of bevacizumab (especially FOLFOX) to oxaliplatin based protocols is beneficial to patients who have not received chemotherapy [52].

First line treatment should also be considered as a potential source of bias. A trial of oxaliplatin based first-line therapy versus maintenance versus observation alone demonstrated: maintenance therapy had no significant effect on prolonging OS. The irinotecan based combination bevacizumab maintenance therapy prolonged OS. However the use of oxaliplatin has cumulative toxicity, especially neurotoxicity. The use of irinotecan based chemotherapy may be more feasible than oxaliplatin based chemotherapy, and more clinical trials on maintenance therapy are needed for further confirmation.

Although this study did not show higher fatal adverse events, a recent meta-analysis involving 16 clinical trials of bevacizumab in solid tumors showed a significant increase in treatment-related mortality (2.5% vs. 1.7%; *P* = 0.01), particularly associated with taxanes and platinum agents (OR = 3.49; 95% CI: 1.82–6.66; incidence, 3.3% vs. 1.0%) [53].

The present study also has certain shortcomings that warrant attention. First, we restricted the search engines and databases to Pubmed, MEDLINE, and the Cochrane Library, which may have limited the number of high-quality rcts searched, thereby weakening the reliability of the results. Second, the included articles were not of high quality and lacked detailed description of allocation concealment and blinding, warranting further studies with rigorous design.Third, the included studies lacked data on indicators such as OS and PFS and could not be included in the comprehensive analysis. Fourth, because the genotypes of Ras and BRAF patients closely related to targeted therapy were not examined in the included studies, the relationship between genotypes and chemotherapy could not be further analyzed, and the potential relationship between genotypes and chemotherapy needs to be further investigated in the future.

### Electronic supplementary material

Below is the link to the electronic supplementary material.

Supplementary Material 1

## Data Availability

The datasets used and/or analysed during the current study available from the corresponding author on reasonable request.
