# Supplementary material for: Effectiveness of bevacizumab in the treatment of metastatic colorectal cancer: a systematic review and meta-analysis
Source: BMC Gastroenterol. 2024 Feb 1;24:58. doi: 10.1186/s12876-024-03134-w (PMC10832121; doi:10.1186/s12876-024-03134-w)

Supplement

Supplementary Table 1. Methodological risk assessment of included studies

| Study ID | Generation of random sequence | Randomized hiding | Blind methods | Withdrawal and loss of interview | Total score |
| --- | --- | --- | --- | --- | --- |
| Salazar 2015^[21]^ | 2 | 1 | 1 | 0 | 4 |
| Salazar 2020^[22]^ | 2 | 1 | 1 | 0 | 4 |
| Qin 2021^[23]^ | 2 | 2 | 2 | 1 | 7 |
| Oki 2019^[24]^ | 2 | 2 | 1 | 1 | 6 |
| Heinemann 2020^[25]^ | 2 | 1 | 1 | 1 | 5 |
| Fischer 2022^[26]^ | 2 | 2 | 1 | 0 | 5 |
| Stathopoulos 2010^[27]^ | 2 | 1 | 1 | 0 | 4 |
| Chibaudel 2020^[28]^ | 2 | 2 | 1 | 0 | 5 |
| Bendell 2019^[29]^ | 2 | 1 | 2 | 1 | 6 |
| Chakravarthy 2020^[30]^ | 2 | 1 | 1 | 1 | 6 |
| Cunningham 2013^[31]^ | 2 | 1 | 1 | 1 | 5 |
| Shitara 2016 | 2 | 1 | 1 | 0 | 4 |
| Snoeren 2017^[33]^ | 2 | 1 | 1 | 0 | 4 |
| Aparicio 2018^[34]^ | 2 | 1 | 1 | 1 | 6 |
| Hurwitz 2004^[35]^ | 2 | 2 | 2 | 0 | 6 |
| Sharf 2022^[36]^ | 2 | 1 | 1 | 0 | 4 |
| Venook 2017^[37]^ | 2 | 1 | 1 | 1 | 6 |
| Passardi 2015^[38]^ | 2 | 1 | 1 | 1 | 5 |
| Dotan 2012^[39]^ | 2 | 1 | 2 | 0 | 5 |
| Moehler 2009^[40]^ | 2 | 1 | 1 | 0 | 4 |
| Cremolini 2016^[41]^ | 2 | 1 | 1 | 0 | 4 |

Supplementary Figure 1. Objective remission rate (ORR) of combined BEV (studies with quality evaluation score>=5 were included only)

**
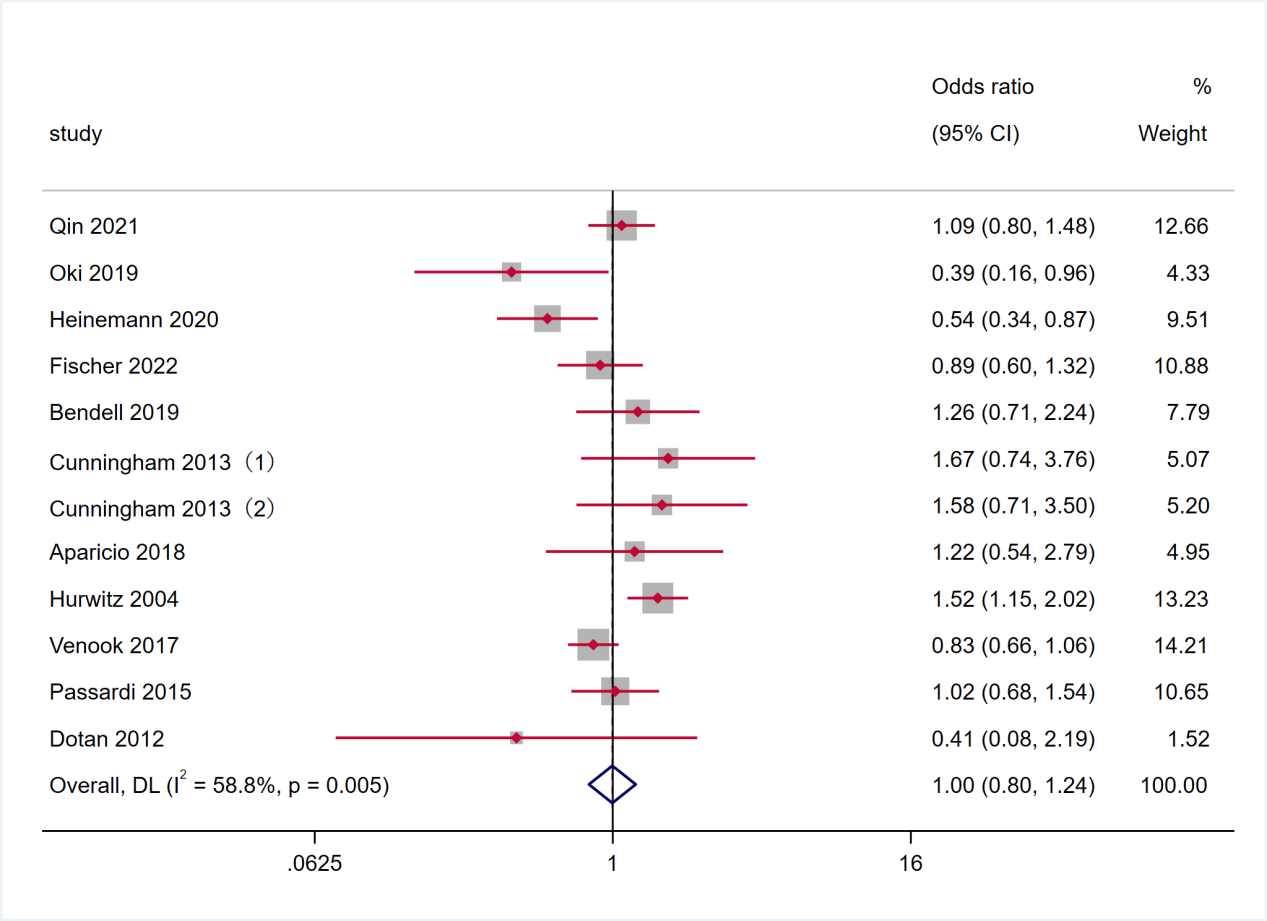
**

Supplementary Figure 2. Disease control rate (DCR) of combined BEV (studies with quality evaluation score>=5 were included only)


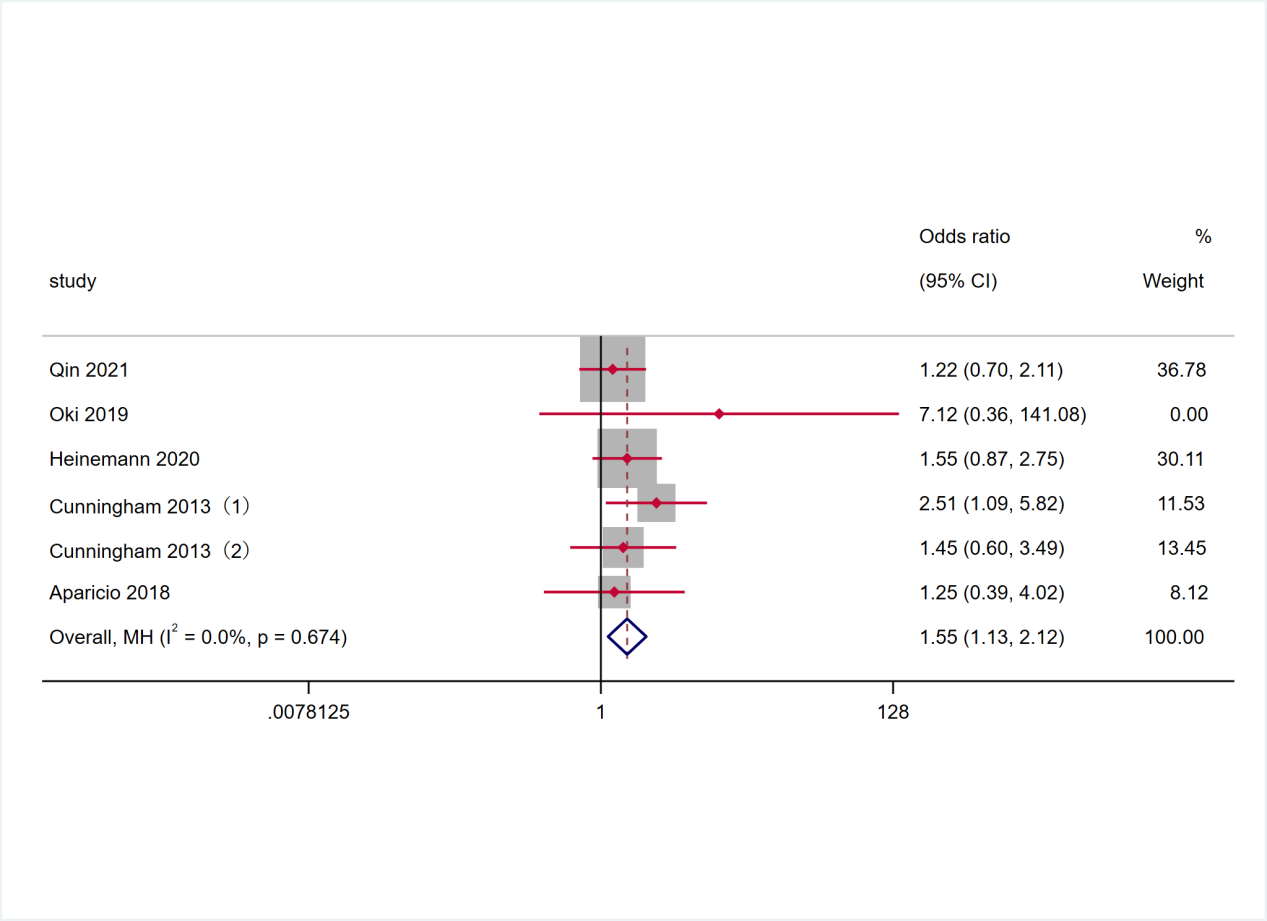


Supplementary Figure 3. Overall survival (OS) of combined BEV (studies with quality evaluation score>=5 were included only)


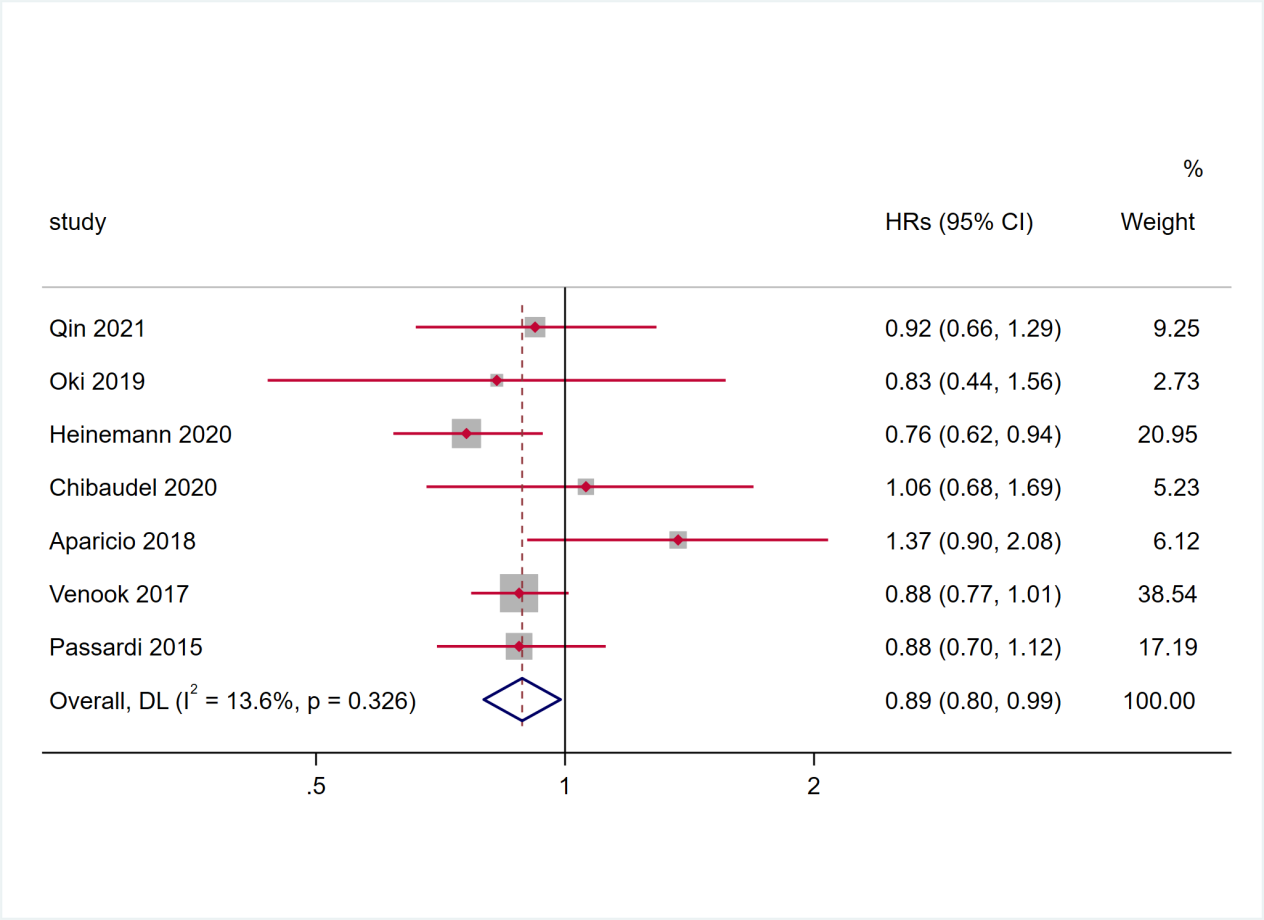


Supplementary Figure 4. Progression-free survival (PFS) of combined BEV (studies with quality evaluation score>=5 were included only)


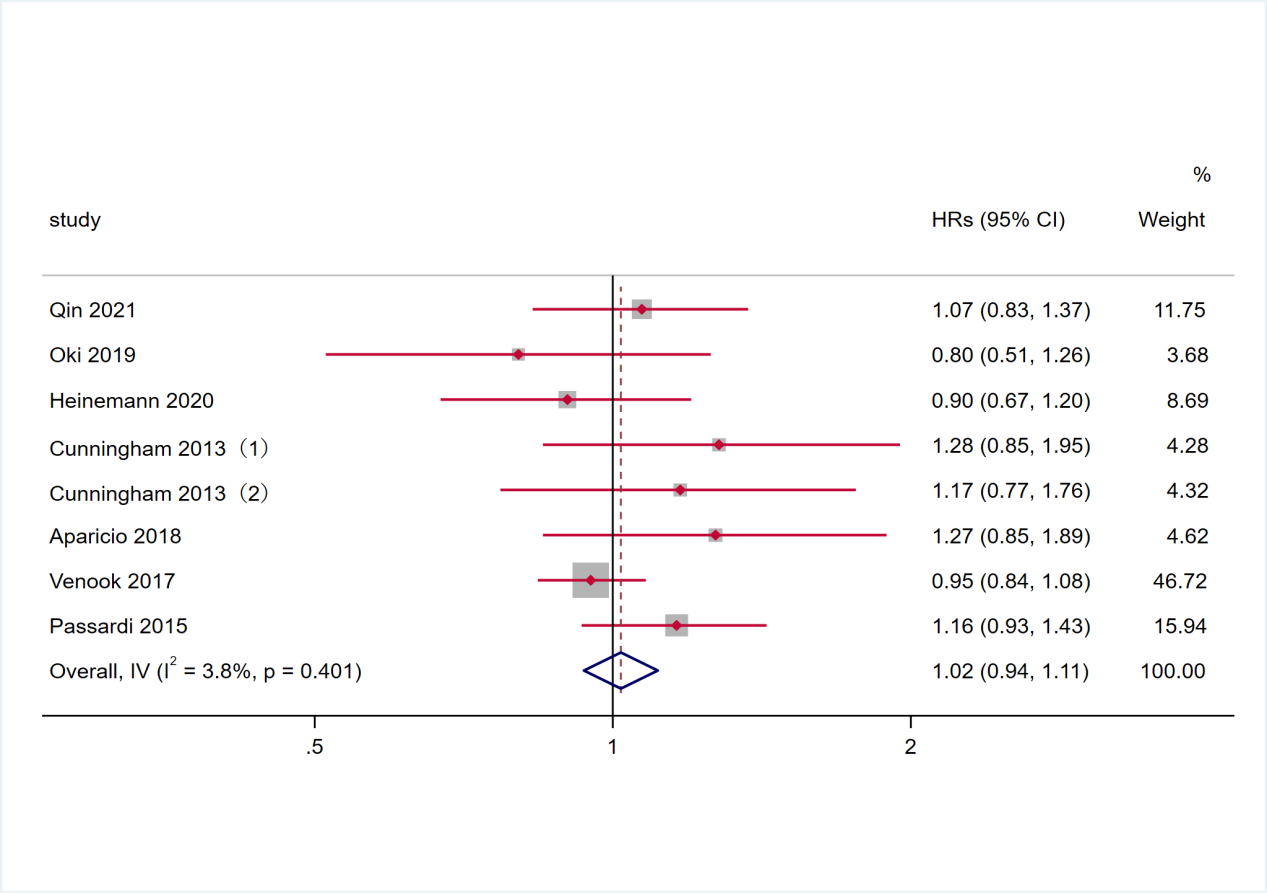

Supplement: Supplementary file 1 — Supplementary Material 1 [file 12876_2024_3134_MOESM1_ESM.docx]
